# Colistimethate sodium is efficacious and safe for the management of sepsis in hematological diseases patients: a retrospective study in China

**DOI:** 10.3389/fcimb.2025.1613414

**Published:** 2025-08-13

**Authors:** Yan Xie, Ziyi Liu, Peiqi Liang, Dong Wang, Qian Li, Meng Gao, Jindan Kong, Depei Wu, Jiajun Qi, Jie Xu, Jianhong Fu

**Affiliations:** ^1^ Department of Hematology, The First Affiliated Hospital of Soochow University, National Clinical Research Center for Hematologic Diseases (Suzhou), Jiangsu Institute of Hematology, Key Laboratory of Thrombosis and Hemostasis of Ministry of Health, Suzhou, Jiangsu, China; ^2^ Intensive Care Medicine Department, The First Affiliated Hospital of Soochow University, Suzhou, Jiangsu, China; ^3^ Education Training Center, The First Affiliated Hospital of Soochow University, Suzhou, Jiangsu, China; ^4^ Center of Clinical Laboratory, The First Affiliated Hospital of Soochow University, Suzhou, Jiangsu, China

**Keywords:** colistimethate sodium, hematological diseases patients, sepsis, effectiveness, safety

## Abstract

**Purpose:**

Patients afflicted with hematological diseases are at an elevated risk of infection, with the potential for the development of sepsis. This study aims to analyze the effectiveness and safety of colistimethate sodium (CMS) in the management of sepsis in Chinese patients with hematological diseases.

**Methods:**

A retrospective study was conducted on hematological diseases patients diagnosed as sepsis and treated with CMS at the First Affiliated Hospital of Soochow University between November 2021 and July 2023. Demographics, clinical presentation, causative organisms, clinical outcomes and adverse events (AEs) were recorded. The univariate and multivariate analysis was conducted to identify predictive factors for clinical effectiveness and renal insufficiency.

**Results:**

A total of 81 hematological patients diagnosed with sepsis were included, the mean (change in sequential organ failure assessment [SOFA] score) ΔSOFA when utilizing CMS was 3.90 ± 2.10. Following CMS treatment, a clinical effectiveness rate was observed in 62.96% of patients, while the bacteriological eradication rate was 60.23%. Additionally, the 28-day all-cause mortality rate was 29.63%. The median length of stay in the ICU and total in-hospital stay was 13.5 and 33 days, respectively. AEs were reported in 16 patients (19.75%), including 15 (18.52%) renal insufficiency, 1 (1.23%) hepatic insufficiency, and 1 (1.23%) rash. The multivariate analysis of clinical effectiveness indicated that a higher Acute Physiology and Chronic Health Evaluation (APACHE) II score, a higher Charlson comorbidity index, the relapse or refractory of hematological diseases, septic shock, and the use of mechanical ventilation was independently associated with a poor clinical effectiveness. The univariate analysis of renal insufficiency demonstrated that allogeneic hematopoietic stem cell transplantation, aplastic anemia, and gastrointestinal infection had a statistically significant impact on renal function.

**Conclusions:**

The findings of our study demonstrated that CMS was an efficacious treatment for sepsis in Chinese patients with hematological diseases, while concurrently exhibiting an acceptable toxicity profile.

## 1 Introduction

Serious infection complications are leading causes for intensive care unit (ICU) admission and mortality in patients with hematological diseases undergoing intensive cytotoxic chemotherapy (Azoulay et al., 2013). Within first year of treatment, 12-27% of patients with hematological diseases develop sepsis due to severe infections, a rate significantly higher than that observed in non-cancer populations (MacPhail et al., 2024). The dysregulated response to infection in patients with sepsis frequently leads to organ dysfunction and circulatory disorders, which can be life-threatening (Font et al., 2020). Estimates indicated a 28-day mortality rate of 67.8% for patients with hematological diseases and septic shock, with a 90-day survival rate of only 19.4% (Manjappachar et al., 2022). Therefore, controlling severe infections and reducing mortality in patients with hematologic disorders pose major clinical challenges.

International guidelines recommend that hematological diseases patients with sepsis using antipseudomonal beta-lactam or carbapenem as empirical antibiotic therapy (Kochanek et al., 2019; Moreno-Sanchez and Gomez-Gomez, 2022). However, the detection of multidrug-resistant (MDR) bacterial pathogens, especially carbapenem-resistant organisms (CRO), poses a significant challenge to the management of sepsis in patients with hematological diseases, often necessitating the reuse of old or non-conventional agents (Hansen et al., 2020). Polymyxins are a peptide antibiotic that targets the outer membrane of Gram-negative bacteria and exhibits significant activity against various drug-resistant bacteria, including CRO pathogens (Nang et al., 2021). Given the paucity of new antibiotics, polymyxins are increasingly being reconsidered as last-resort antibiotics for MDR bacterial pathogens infection.

**Figure 1:**
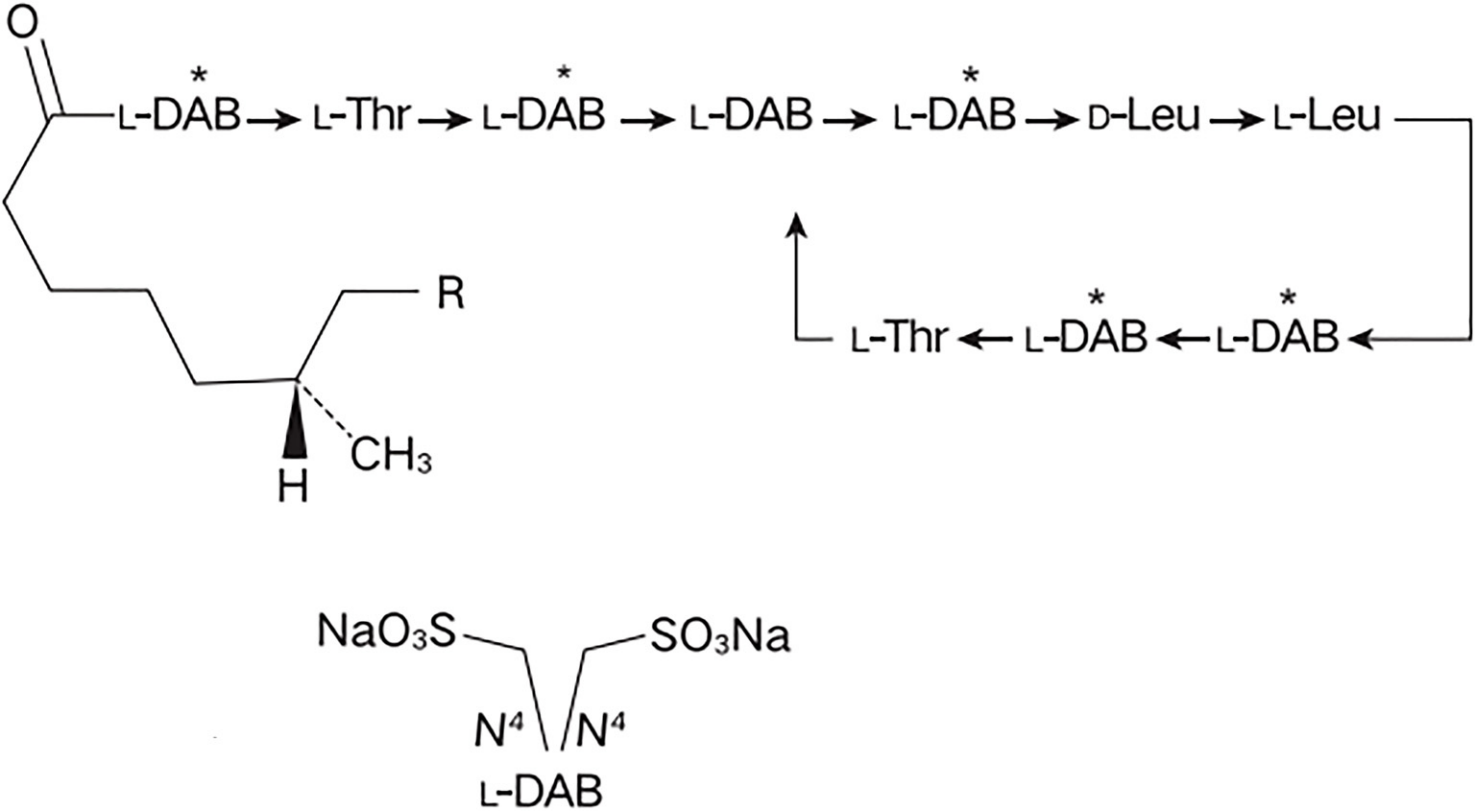
The chemical structure of colistimethate sodium. L-DAB, Levogyre-Diaminobutyric acid; L-Thr, Levogyre-Threonine; D-Leu, Dextrogyre- Leucine; R, in polymyxin E1, R = CH_3_, in polymyxin E2, R = H.

Colistimethate sodium (CMS) is a polyanionic inactive prodrug formed by the reaction of colistin with formaldehyde and sodium bisulfite (**Figure 1**). This prodrug is gradually hydrolyzed and transformed into colistin base and various inactive methanesulfonated compounds in aqueous media and biological fluids, providing a relatively long-lasting antibacterial effect while significantly reducing the risk of kidney injury at the initial stage of administration (Poirel et al., 2017; Xu et al., 2023). In addition to its direct antibacterial activity, CMS also exhibits potent immunomodulatory properties which might be beneficial for sepsis patients with cytokine dysregulation (Matzneller et al., 2017). CMS has been adopted by hospitals worldwide and as salvage therapy for otherwise untreatable gram-negative infections (Tsuji et al., 2019). Studies have shown that CMS is effective in treating severe infections caused by multidrug-resistant *Pseudomonas aeruginosa* (MDR-PA) in patients with hematological diseases, with 76.9% of patients achieved resolution of infection (Durakovic et al., 2011). In ICU sepsis patients, CMS demonstrated favorable clinical effectiveness with an observed treatment response rate of 82.1% (Dalfino et al., 2012). Particularly, CMS also demonstrates well tolerability in patients with hematological diseases, no serious adverse events (AEs) was observed after high dose CMS−based therapy (Grignolo et al., 2017). However, due to the late launch of CMS in China, clinical evidence is still limited, especially in the treatment of sepsis in hematological diseases patients.

This study retrospectively analyzed the effectiveness and safety of CMS in the management of Chinese hematological diseases patients with sepsis, aiming to provide more evidence for the clinical application of CMS and achieve the precise treatment of sepsis in patients with hematological diseases.

## 2 Materials and methods

### 2.1 Study design and population

This study was a single-center, retrospective study conducted at the First Affiliated Hospital of Soochow University in Jiangsu, China. Hospitalized patients with hematological diseases who were diagnosed as sepsis and treated with CMS between November 2021 and July 2023 were included to analyze the clinical effectiveness and safety. According to the Sepsis-3 criteria, patients with suspected infection demonstrating an increase of ≥2 points in the Sequential Organ Failure Assessment (SOFA) score from baseline, and typically requiring ICU admission, are diagnosed with sepsis. ICU admission criteria: 1) Persistent deterioration in SOFA score; 2) Need for vasoactive drugs to maintain blood pressure; 3) Respiratory failure requiring mechanical ventilation. The demographic, clinical, laboratory and microbiological characteristics of each enrolled patient were collected at baseline (before the onset of sepsis), administration and follow-up period (28 ± 1 day after the start of administration). The study was conducted in accordance with the Declaration of Helsinki and approved by the First Affiliated Hospital of Soochow University Medical Ethics Committee (2023-452).

### 2.2 Isolation of strains and antimicrobial sensitivity testing

The isolation, cultivation, and identification of strains were carried out in accordance with the national clinical laboratory practice. The samples were collected within one hour of sepsis and prior to CMS administration. The mass spectrometry identification, VITEK MS automatic rapid microbial mass spectrometry detection system, the VITEK AST GN67 and XN04 card (bioMérieux, France) was used for strain identification and antimicrobial sensitivity testing. The minimum inhibitory concentrations (MICs) of colistin for causative organisms were determined by the broth micro-dilution (bio-kont, China), classified using the European Committee for Drug Allergy Testing (EUCAST 2020) standards. Other drugs MICs were interpreted based on the American Clinical and Laboratory Standards Institute (CLSI) M100–2019 standards.

### 2.3 CMS administration

All enrolled patients received initial empiric or salvage therapy of CMS (Chia Tai Tianqing Pharmaceutical Group Co., Ltd, China). On the basis of local antimicrobial resistance monitoring data and renal function of patients, the initial empirical treatment of CMS was recommended for patients who have progressed to sepsis after receiving a broad-spectrum β-lactam or carbapenems, particularly in high-risk MDR populations such as those who have undergone allogeneic hematopoietic stem cell transplantation (allo-HSCT). Furthermore, in cases where pathogen infections have been demonstrated to be sensitive to CMS *in vitro* drug sensitivity tests, CMS was utilized as a targeted therapeutic agent. Typically, CMS is administered at a dose of 2.5 to 5 mg/kg/day intravenously. The median time to symptom improvement using CMS in this study was 6.00 days. The specific dosage was adjusted by clinical physicians based on the renal function of each patient, and the maximum daily dose is not more than 5 mg/kg. The differences between empirical therapy and targeted therapy in terms of applicable conditions and basis for decision were shown in **Supplementary Table S1**.

### 2.4 Clinical outcomes

The primary outcome was the clinical effectiveness evaluated at the conclusion of administration. Clinical effectiveness was characterized by the fulfillment of all key criteria: 1) patient survival, 2) significant improvement or complete resolution of infection-related clinical symptoms/signs, and 3) normalization of inflammatory biomarkers (including C-reactive protein [CRP] and procalcitonin [PCT]) or a reduction of at least 50% from baseline levels, along with at least one supportive criterion: 1) hemodynamic stability without vasopressor support, or 2) microbiologically confirmed eradication (defined as negative cultures) or presumed eradication. The secondary outcomes included: 1) Bacteriological eradication at the end of administration, defined as the proportion of patients with eradication and presumed eradication in the patients with identified pathogenic bacteria. The bacteriological eradication was defined as the failure to cultivate pathogenic bacteria from the original infected site of the specimen after treatment, presumed eradication was defined as the disappearance of symptoms and signs makes it impossible to obtain cultivable materials (such as sputum, skin pus, or secretions), or the method of obtaining specimens is too invasive for rehabilitation patients. 2) 28-day all-cause mortality, which was defined as the proportion of the total number of deaths caused by various reasons within 28 days after the start of administration in the total population. 3) The length of ICU stay and total in-hospital stay. 4) The incidence of renal insufficiency, which was defined using RIFLE criteria. The ‘‘Risk,’’ ‘‘Injury,’’ and ‘‘Failure’’ of the RIFLE criteria were based on peak serum creatinine (Scr) values during CMS administration.

### 2.5 Safety analysis

The safety was evaluated based on AEs that occurred during the administration and follow-up. The occurrence of renal insufficiency based on the RIFLE criteria was recorded. The AEs related to CMS treatment was listed and corresponding incidence rates were calculated.

### 2.6 Statistical analysis

All statistical analyses were performed with SPSS version 16.0 (SPSS Inc., Chicago, IL). The descriptive statistics was used to assess the baseline characteristics of patients. Continuous variables were represented by mean ± standard deviation (SD) or median (interquartile range, IQR), and categorical variables were represented by the corresponding number of cases and percentage. Clopper-Pearson method was used to calculate the 95% confidence interval of clinical effectiveness, bacteriological eradication, and other indicators. The univariate and multivariate analysis of clinical effectiveness and renal insufficiency was conducted, and two independent sample T-tests was used if the continuous variables follow a normal distribution; On the contrary, non-parametric Mann Whitney U test was used. The chi-square test or Fisher’s exact test was used for categorical variables.

## 3 Results

### 3.1 Baseline characteristics

A total of 81 hematological patients diagnosed with sepsis were included, the demographic and clinical characteristics before the onset of sepsis were shown in **Table 1**. The mean age of patients was 45.21 ± 14.55 years, and 61.73% were male. The most common underlying conditions in hematological was acute leukemia (66.67%), followed by lymphoma (19.75%) and aplastic anemia (AA, 6.17%). 32.1% of patients received allo-HSCT. 29.63% of patients with past medical history, and the three most common diseases were hypertension (14/24, 58.33%), diabetes (8/24, 33.33%), and hepatitis (5/24, 20.83%). 45.68% of patients experienced septic shock, and 72.84% of patients developed neutropenia before the infection. Among these neutropenia patients, 58 patients had neutrophil counts below 0.5×10^9^/L, including 48 patients meeting the criteria for severe neutropenia (<0.1×10^9^/L). The median duration of neutropenia before the CMS therapy in this cohort was 12 days. The mean acute physiology and chronic health evaluation (APACHE) II score and Charlson comorbidity index of patient was 13.54 ± 5.80 and 3.53 ± 1.77, respectively. The mean ΔSOFA score when utilizing CMS was 3.90 ± 2.10. Sixty-three patients (77.78%) had a documented causative pathogen of sepsis, of which carbapenem-resistant *Klebsiella pneumoniae* (30.16%) was the most common causative organism, followed by carbapenem-resistant *Pseudomonas aeruginosa* (12.70%). The median MICs of colistin against causative organisms were 1 µg/mL. Lung infection (62.96%) was the most common infection, followed by bloodstream infection (48.15%). 20.99% of patients underwent mechanical ventilation and 45.68% of patients received vasoactive agent. None of the patients received prophylactic antibiotic therapy. In view of the severe sepsis, all patients were treated with an anti-infective therapy containing CMS. 79.01% of patients received CMS empirical therapy, while 24.69% of patients were adjusted to CMS targeted therapy after drug susceptibility results.

**Table 1:** The demographic and clinical characteristics of patients.

Variables	Total (n = 81)
Age, years, mean ± SD	45.21 ± 14.55
Gender, male, no. (%)	50 (61.73)
Underlying conditions in hematological, no. (%)	
Malignancies	
Acute leukemia	54 (66.67)
Lymphoma	16 (19.75)
AA	5 (6.17)
MDS	2 (2.47)
Others	4 (4.94)
Diagnosis of hematological diseases, no. (%)	
Newly diagnosed	29 (35.80)
Remission	15 (18.52)
Relapse or refractory	37 (45.68)
Allo-HSCT, no. (%)	26 (32.10)
Past medical history, no. (%)	24 (29.63)
Hypertension	14 (58.33)
Diabetes	8 (33.33)
Hepatitis	5 (20.83)
Others^*^	4 (16.67)
Neutropenia before the infection, no. (%)	59 (72.84)
<0.5×10^9^/L	59 (100%)
<0.1×10^9^/L	48 (81.36)
Duration of neutropenia before CMS therapy, days, median (IQR)	12.00 (5.00, 23.50)
Septic shock, no. (%)	37 (45.68)
APACHE II score, mean ± SD	13.54 ± 5.80
Charlson comorbidity index, mean ± SD	3.53 ± 1.77
Procalcitonin^#^, ng/mL, mean ± SD	16.69 ± 25.79
ΔSOFA score when utilizing CMS, mean ± SD	3.90 ± 2.10
Detection of causative organisms, no. (%)	63 (77.78)
Main causative organisms, no. (%)	
Carbapenem-resistant *Klebsiella pneumonia* (median colistin MICs 1 µg/mL)	19 (30.16)
Carbapenem-resistant *Pseudomonas aeruginosa* (median colistin MICs 1 µg/mL)	8 (12.70)
Carbapenem-resistant *Enterobacteriaceae* (median colistin MICs 1 µg/mL)	4 (3.65)
Carbapenem-resistant *Acinetobacter baumannii* (median colistin MICs 1 µg/mL)	3 (4.76)
Site of infection, no. (%)	
Lung	51 (62.96)
Bloodstream	39 (48.15)
Skin soft-tissue	15 (18.52)
Digestive tract	11 (13.58)
Others	2 (2.47)
Mechanical ventilation, no. (%)	17 (20.99)
Vasoactive agent, no. (%)	37 (45.68)
Colistin MICs, µg/mL, median (min-max) (min-max)	1 (0.25, 2)
CMS therapy, no. (%)	
Empirical therapy	64 (79.01)
Targeted therapy	20 (24.69)

Data are presented as mean ± SD, no. (%), median (IQR), where no. is the total number of patients with available data.

^*^The others past medical history include 2 heart diseases, 1 chronic obstructive pulmonary disease and 1 other malignancy.

^#^The laboratory reference range for procalcitonin is 0-0.5 ng/mL.

MDS, myelodysplastic syndromes; AA, aplastic anemia; Allo-HSCT, allogeneic hematopoietic stem cell transplantation; APACHE II, acute physiology and chronic health evaluation II; ΔSOFA, change in Sequential Organ Failure Assessment score from baseline; MICs, minimum inhibitory concentrations; CMS, Colistimethate sodium.

**Figure 2:**
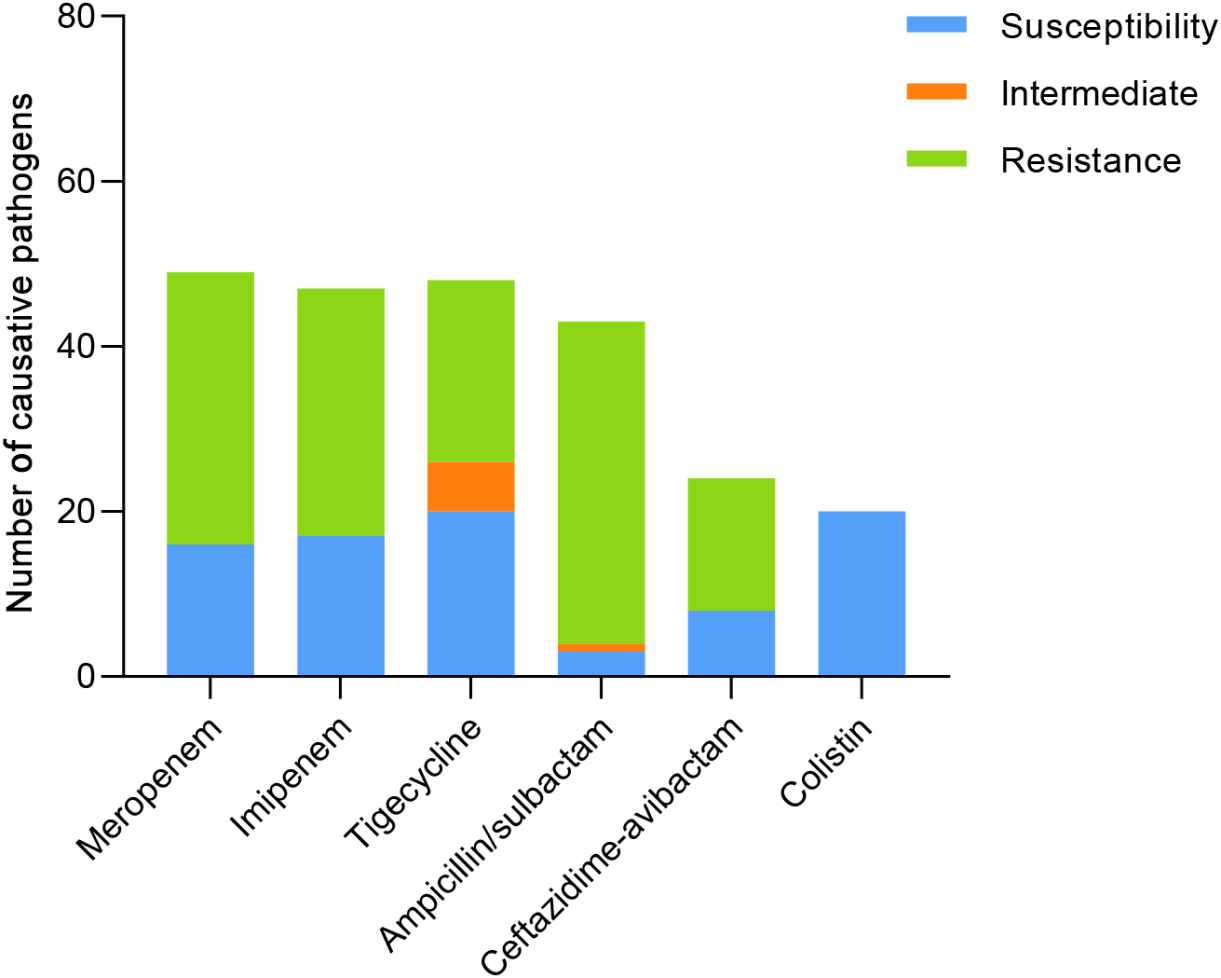
Susceptibility of causative pathogens to antimicrobial agents.

The resistance of causative pathogens to meropenem and imipenem was 66% and 63.83%, respectively. 41.67% of causative pathogens were susceptible to tigecycline, while the antimicrobial nonsusceptibility to ampicillin/sulbactam and ceftazidime-avibactam was 93.03% and 66.67%. Notably, 100% of causative pathogens were susceptible to colistin, with MICs of ≤ 2.0 µg/mL (**Table 2**; **Figure 2**).

**Table 2:** Susceptibility of causative pathogens to antimicrobial agents.

Antimicrobial Agents	Tested, no.	Susceptibility no. (%)	Intermediate no. (%)	Resistance no. (%)
Meropenem	50	16 (32.00)	0	33(66.00)
Imipenem	47	17 (36.17)	0	30 (63.83)
Tigecycline	48	20 (41.67)	6 (12.50)	22(45.83)
Ampicillin/sulbactam	43	3 (6.98)	1 (2.33)	39 (90.70)
Ceftazidime-avibactam	24	8 (33.33)	0	16 (66.67)
Colistin	20	20 (100.00)	0	0

### 3.2 Clinical outcomes and safety

**Figure 3:**
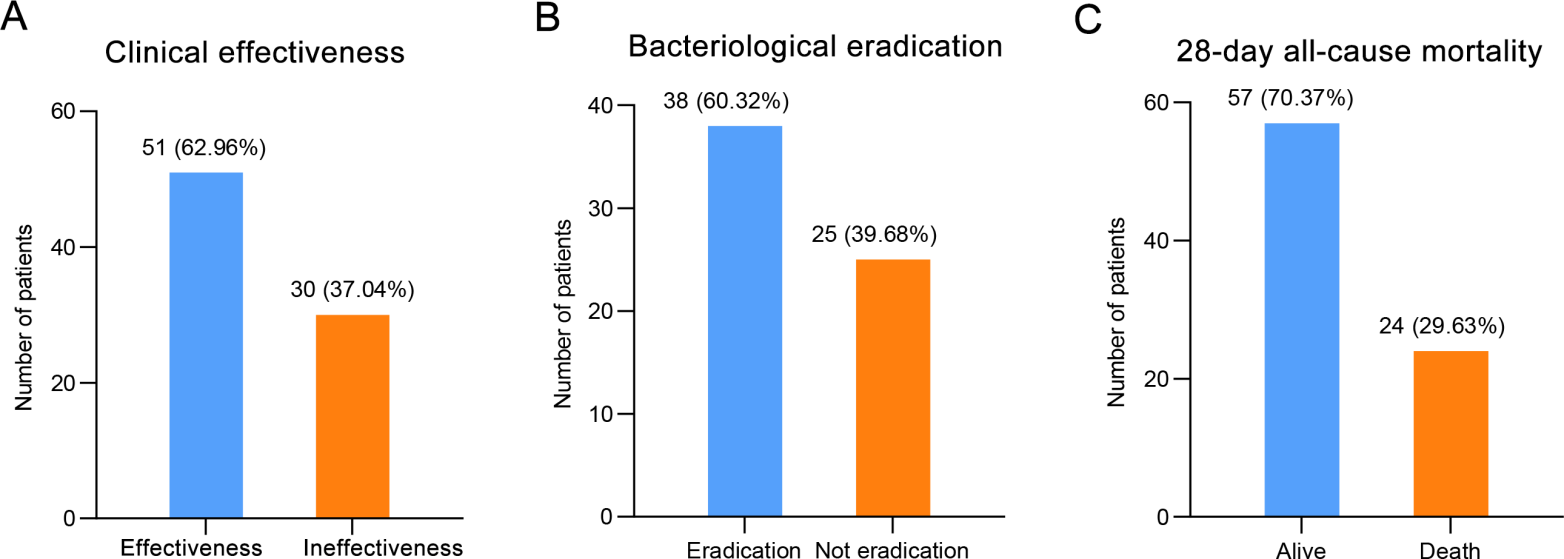
The clinical outcomes of patients treated with CMS, including **(A)** clinical effectiveness, **(B)** bacteriological eradication, and **(C)** 28-day all-cause mortality.

Clinical effectiveness was seen in 62.96% (95% CI: 51.51-73.44) of patients and clinical ineffective was seen in 37.04% of patients (**Table 3**; **Figure 3**). Bacteriological eradication was obtained in 38 patients (60.32%, 95% CI: 47.20-72.43), with persistent infection observed in 13 patients (20.63%). Among the 59 patients who developed neutropenia before infection, 47 patients were detected with pathogenic bacteria, with a bacteriological eradication rate of 59.57% (28/47). The bacteriological eradication rate of patients with severe neutropenia was 53.66% (22/41), which was slightly lower than that of all neutropenia patients. Overall, the 28-day all-cause mortality was 29.63% (95% CI: 19.99-40.81). The median length of ICU and total in-hospital stays was 13.5 (95% CI: 12.00-22.50) and 33 (95% CI: 31.50-38.00) days, respectively. The median time to symptom improvement was 6 (95% CI: 5.50-8.50) days.

**Table 3:** Clinical outcomes of patients treated with CMS.

Variables	No. (%) or median (min, max)	95% CI
Primary outcome		
Clinical effectiveness	51 (62.96)	51.51, 73.44
Secondary outcomes		
Bacteriological eradication^*^	38 (60.32)	47.20, 72.43
28-day all-cause mortality	24 (29.63)	19.99, 40.81
Length of ICU stay, days	13.50 (1.00, 79.00)	12.00, 22.50
Length of total in-hospital stay, days	33.00 (4.00, 98.00)	31.50, 38.00
Time to symptom improvement, days	6.00 (2.00, 39.00)	5.50, 8.50
Renal insufficiency	15 (18.52)	10.15, 28.70

^*^Apart from the bacteriological eradication which was calculated among the 63 patients who had the pathogenic bacteria detected, all other outcomes were calculated in the entire population.

Furthermore, a subgroup analysis was conducted to evaluate the effectiveness of different CMS therapy regimens (**Table 4**). The clinical effectiveness rate and the bacteriological eradication rate of CMS targeted therapy were both 66.67% (95% CI: 43.03-85.41). The clinical effectiveness rate and the bacteriological eradication rate of CMS empirical therapy was 60.66% (95% CI: 47.31-72.93) and 55.81% (95% CI: 39.88-70.92), respectively, which was comparable to the effectiveness of targeted therapy. However, the median length of total in-hospital stay in the targeted therapy group was slightly longer than that in the empirical treatment group (35.86, 95% CI: 29.53-42.19 vs. 33.00, 95% CI: 30.50-38.00). Notably, only one patient received CMS monotherapy, and achieved clinical effectiveness and bacteriological eradication during the 32-day hospitalization period. Among different CMS combination therapy regimens, the effectiveness of CMS combined with amikacin and aztreonam was higher than that of other combined regimens, with a clinical effectiveness rate of 81.82% (9/11, 95% CI: 48.22-97.72), and a bacteriological eradication of 80.00% (8/10, 95% CI: 44.39-97.48).

**Table 4:** Subgroup analysis of clinical outcomes in patients treated with CMS.

Type of therapy	Clinical effectiveness (n=81), no. (%), (95%CI)	Bacteriological eradication^*^ (n=63), no. (%), (95%CI)	Length of total in-hospital stay, (n=81), days, median, (95%CI)
CMS therapy			
Empirical therapy	37 (60.66), (47.31, 72.93)	24 (55.81), (39.88, 70.92)	33.00, (30.50, 38.00)
Targeted therapy	14 (66.67), (43.03, 85.41)	14 (66.67), (43.03, 85.41)	35.86, (29.53, 42.19)
CMS monotherapy	1 (100.00), (2.50,100.00)	1 (100.00), (2.50,100.00)	32.00, (32.00, 32.00)
CMS combination therapy^#^			
Penicillin preparations	4 (57.14), (18.41, 90.10)	3 (60.00), (14.66, 94.72)	38.57, (31.93, 45.22)
Carbapenems	21 (53.85), (37.18, 69.91)	15 (51.72), (32.53, 70.55)	33.00, (28.00, 38.00)
Tigecycline	12 (60.00), (36.05, 80.88)	9 (52.94), (27.81, 77.02)	35.00, (31.50, 42.00)
Third-generation Cephalosporins	7 (77.78), (39.99, 97.19)	5 (71.43), (29.04, 96.33)	38.56, (21.71, 55.41)
Ceftazidime-avibactam	6 (66.67), (29.93, 92.51)	5 (62.50), (24.49,91.48)	31.50, (26.00, 51.50)
Others	9 (81.82), (48.22, 97.72)	8 (80.00), (44.39, 97.48)	41.27, (30.47, 52.07)

^*^Apart from the bacteriological eradication which was calculated among the 63 patients who had the pathogenic bacteria detected, all other outcomes were calculated in the entire population.

^#^The penicillin preparation is piperacillin-tazobactam. The carbapenem drugs include meropenem, imipenem and biapenem. Other drugs include amikacin and aztreonam.

Among the 81 patients, AEs were reported in 16 patients (**Table 5**). Renal insufficiency was reported in 18.52% (15/81) patients, which was the most frequent adverse event. According to the RIFLE criteria, patients with renal insufficiency were classified as risk in 5 (33.33%) patients, injury in 9 (60%) patients, and failure in 1 (6.67%) patient. The dosage of CMS was adjusted based on the SCr clearance rate for patients with renal insufficiency. One patient who was in the “Failure” stage and 4 patients who did not meet the “Failure” criteria but whose Scr levels were showing a rising trend were discontinued from using CMS. It is worth noting that none of the patients progressed to require continuous renal replacement therapy (RRT) for at least 4 weeks. Additionally, CMS exhibited favorable tolerance with 1 (1.23%) hepatic insufficiency and 1 (1.23%) rash.

**Table 5:** Adverse events possibly or probably related to the use of CMS.

Adverse events, no. (%)	Total (n = 81)
Renal insufficiency (RIFLE criteria)	15 (18.52)
Risk (R) SCr increase ≥50% or eGFR decrease >25%	5 (33.33)
Injury (I) SCr increase ≥100% or eGFR decrease >50%	9 (60.00)
Failure (F) SCr increase ≥200% or eGFR decrease 75%	1 (6.67)
Loss (L) Complete loss of kidney function (need for RRT) >4 weeks	0
ESKD (E) End stage kidney disease (need for RRT) >3 months	0
Hepatic insufficiency	1 (1.23)
Rashes	1 (1.23)

CMS, Colistimethate sodium; SCr, serum creatinine; RRT renal replacement therapy.

### 3.3 Predictors of clinical ineffective and renal insufficiency

The univariate analysis of clinical effectiveness indicated that several factors such as age, past medical history, APACHE II score, Charlson comorbidity index score, ΔSOFA score when utilizing CMS, hematological diagnosis, septic shock, requirement for mechanical ventilation significantly influence clinical outcomes. The multivariate analysis of clinical effectiveness revealed that a higher APACHE II score, a higher Charlson comorbidity index, relapse or refractory of hematological diseases, septic shock, and the administration of mechanical ventilation were independently associated with poor clinical effectiveness (**Table 6**). The univariate analysis of renal insufficiency demonstrated that allo-HSCT, aplastic anemia, and digestive tract infection had a statistically significant impact on renal function in hematological diseases patients (**Table 7**).

**Table 6:** Univariate and multivariate analysis of factors associated with clinical outcome.

Variables	Clinical effectiveness	Clinical ineffectiveness	Univariate analysis		Multivariate analysis	
	(n = 51)	(n = 30)	OR (95%CI)	*P* value	OR (95%CI)	*P* value
Age, years, mean ± SD	42.67 ± 13.83	49.53 ± 14.95	0.97 (0.93, 0.99)	**0.043**	1.01 (0.96, 1.06)	0.829
Gender, no. (%)			1.40 (0.55, 3.68)	0.484		
Male	21 (41.18)	10 (33.33)				
Female	30 (58.82)	20 (66.67)				
Underlying conditions in hematological, no. (%)						
Acute leukemia	36 (70.59)	18 (60.00)	1.60 (0.62, 4.15)	0.331		
Lymphoma	11 (21.57)	5 (16.67)	1.38 (0.44, 4.79)	0.593		
AA	3 (5.88)	2 (6.67)	0.88 (0.14, 6.95)	0.887		
MDS	0 (0.00)	2 (6.67)	/	/		
Diagnosis of hematological diseases, no. (%)						
Newly diagnosed	23 (45.10)	6 (20.00)	3.29 (1.20, 10.10)	**0.027**	**/**	/
Remission	9 (17.65)	6 (20.00)	0.86 (0.28, 2.83)	0.792	0.22 (0.03, 1.51)	0.135
Relapse or refractory	19 (37.25)	18 (60.00)	0.39 (0.15, 0.99)	0.050	0.14 (0.02, 0.64)	**0.019**
Neutropenia before the infection, no. (%)	37 (72.55)	22 (73.33)	0.96 (0.34, 2.62)	0.939		
Allo-HSCT, no. (%)	17 (33.33)	9 (30.00)	1.17 (0.45, 3.18)	0.756		
Past medical history^*^, no. (%)	11 (21.57)	13 (43.33)	0.36 (0.13, 0.96)	**0.041**	0.56 (0.14, 2.18)	0.399
Septic shock, no. (%)	18 (35.29)	19 (63.33)	0.316(0.120,0.794)	0.016	0.16 (0.02, 0.87)	**0.042**
APACHEII score, mean ± SD	12.37 ± 5.74	15.53 ± 5.42	0.90 (0.82, 0.98)	**0.023**	0.88 (0.78, 0.99)	**0.038**
CCI score, mean ± SD	3.06 ± 1.64	4.33 ± 1.71	0.64 (0.47, 0.85)	**0.003**	0.61 (0.38, 0.91)	**0.021**
Procalcitonin^#^, ng/mL, mean ± SD	15.88 ± 27.84	18.07 ± 22.23	0.99 (0.98, 1.02)	0.711		
ΔSOFA score when using CMS, mean ± SD	3.47 ± 1.93	4.63 ± 2.20	0.77 (0.60, 0.95)	**0.020**	1.41 (0.88, 2.42)	0.174
Mechanical ventilation, no. (%)	4 (7.84)	13 (43.33)	0.11 (0.03, 0.36)	**0.001**	0.12 (0.02, 0.60)	**0.016**
Site of infection, no. (%)						
Lung	29 (56.86)	22 (73.33)	0.48 (0.17, 1.25)	0.142		
Bloodstream	21 (41.18)	18 (60.00)	0.47 (0.18, 1.16)	0.104		
Skin soft-tissue	10 (19.61)	5 (16.67)	1.22 (0.39, 4.29)	0.742		
Digestive tract	7 (13.73)	4 (13.33)	1.43 (0.36, 7.08)	0.624		

^*^The past medical history includes diabetes, hypertension, heart disease, hepatitis, chronic obstructive pulmonary disease and other malignancies.

^#^The laboratory reference range for procalcitonin is 0-0.5 ng/mL.

MDS, myelodysplastic syndromes; AA, aplastic anemia; Allo-HSCT, allogeneic hematopoietic stem cell transplantation; APACHE, acute physiology and chronic health evaluation; CCI, Charlson comorbidity index; GCS, Glasgow Coma Scale; ΔSOFA, change in Sequential Organ Failure Assessment score from baseline.

Bold values indicate P < 0.05.

**Table 7:** Univariate analysis of factors associated with renal insufficiency.

Variables	Renal insufficiency	Normal renal function	Univariate analysis	
	(n = 15)	(n= 66)	OR (95%CI)	*P* value
Age, years, mean ± SD	44.87 ears, m	45.29 ± 15.12	0.99 (0.96, 1.04)	0.919
Gender, no. (%)			2.14 (0.69, 6.83)	0.189
Male	8 (53.33)	23 (34.85)		
Female	7 (46.67)	43 (65.15)		
Underlying conditions in hematological, no. (%)				
Acute leukemia	7 (46.67)	47 (71.21)	1.02 (0.21, 3.80)	0.979
Lymphoma	3 (20.00)	13 (19.70)	0.35 (0.11, 1.11)	0.075
AA	4 (26.67)	1 (1.52)	23.63 (3.15, 486.39)	**0.007**
MDS	1 (6.67)	1 (1.52)	4.64 (0.18, 122.27)	0.288
Diagnosis of hematological diseases, no. (%)				
Newly diagnosed	6 (40.00)	23 (34.85)	1.25 (0.38, 3.90)	0.708
Remission	3 (20.00)	12 (18.18)	1.13 (0.23, 4.24)	0.870
Relapse or refractory	6 (40.00)	31 (46.97)	0.75 (0.23, 2.33)	0.625
Neutropenia before the infection, no. (%)	10 (66.67)	49 (74.24)	0.69 (0.21, 2.49)	0.553
Allo-HSCT, no. (%)	9 (60.00)	17 (25.76)	4.32 (1.36, 14.67)	**0.014**
Past medical history^*^, no. (%)	5 (33.33)	19 (28.79)	1.24 (0.35, 3.99)	0.728
Septic shock, no. (%)	6 (40.00)	31 (46.97)	0.75 (0.23, 2.33)	0.625
APACHEII score, mean ± SD	14.73 ± 5.46	13.27 ± 4.26	1.04 (0.95, 1.15)	0.379
CCI score, mean ± SD	3.52 ± 1.79	3.55 ± 1.77	0.78 (0.51, 1.11)	0.201
Procalcitonin^#,^ ng/mL, mean ± SD	20.33 ± 26.44	15.86 ± 25.77	1.01 (0.98,1.03)	0.545
ΔSOFA score when using CMS, mean ± SD	3.83 ± 2.10	3.93 ± 2.10	0.85 (0.60, 1.13)	0.309
Mechanical ventilation, no. (%)	3 (20.00)	14 (21.21)	0.93 (0.19, 3.43)	0.917
Site of infection, no. (%)				
Lung	9 (60.00)	42 (63.64)	0.86 (0.28,2.83)	0.792
Bloodstream	6 (40.00)	33 (50.00)	0.67 (0.20, 2.06)	0.486
Skin soft-tissue	1 (6.67)	14 (21.21)	0.27 (0.01, 1.50)	0.218
Digestive tract	6 (40.00)	5 (7.58)	8.13 (2.06, 34.10)	**0.003**

^*^The past medical history includes diabetes, hypertension, heart disease, hepatitis, chronic obstructive pulmonary disease and other malignancies.

^#^The laboratory reference range for procalcitonin is 0-0.5 ng/mL.

MDS, myelodysplastic syndromes; AA, aplastic anemia; Allo-HSCT, allogeneic hematopoietic stem cell transplantation; APACHE, acute physiology and chronic health evaluation; CCI, Charlson comorbidity index; GCS, Glasgow Coma Scale; ΔSOFA, change in Sequential Organ Failure Assessment score from baseline.

Bold values indicate P < 0.05.

## 4 Discussion

With the increasingly severe situation of drug resistance and the emergence of multidrug-resistant and extensively resistant Gram-negative bacteria, polymyxins have returned to clinical as the last-resort antibiotics. However, due to its relatively late launch in China, the clinical application of CMS was relatively lacking, especially in Chinese hematological patients with sepsis, which have not been reported yet. Our findings for the first time demonstrated that anti-infective therapy containing CMS was effective and safe in Chinese patients with hematological diseases, providing strong clinical evidence of medication for sepsis in hematological diseases patients.

CRO infections pose a significant challenge in Chinese patients with hematological diseases due to their high infection rates and limited treatment options. The findings of this study indicate that a proportion of infected patients exhibit a progression to sepsis, despite administration of broad-spectrum β-lactam or carbapenem drugs. Furthermore, carbapenem-resistant enterobacteriaceae (CRE) exhibits a higher rate of resistance to alternative drugs, such as tigecycline and ceftazidime-avibactam (**Table 2**). In contrast, CMS demonstrates noteworthy antibacterial activity against the majority of carbapenem-resistant Gram-negative bacteria, exhibiting a clinical effectiveness of approximately 60% in multidrug-resistant Gram-negative bacterial infections (Kagami et al., 2021). Consequently, despite the international guidelines being cautious with regard to the application of CMS in CRE infection, taking into consideration the substantial difference between the epidemiology of CRO infection in China and international data, as well as the availability of existing drugs, CMS can be a promising choice for septic patients with hematological diseases who have high carbapenem resistance and limited alternative drugs. It is noteworthy that the bacteriological eradication rate of 60.23% in this study is consistent with the results in multidrug-resistant Gram-negative bacteria (Kagami et al., 2021), thereby suggesting the effectiveness of CMS in this specific populations.

In this retrospective study analyzing the records of 81 patients, CMS presented as an effective antimicrobial agent, with a clinical effectiveness rate of 62.96%, a bacteriological eradication rate of 60.23% and 28-day all-cause mortality of 29.63%. The median length of ICU stay and total in-hospital stay was 13.5 and 33 days, respectively. Additionally, CMS has shown a good safety profile for the treatment of sepsis. Renal insufficiency occurred in 18.52% of patients, and the renal impairment were mild, no case of permanent kidney function loss occurred. The emergence of antibiotic resistance and the lack of new antibiotics have increased the use of CMS. Previous studies have demonstrated that in patients receiving CMS treatment for MDR-Gram-negative bacteria, the clinical effective rates are 50% - 86%, the bacteriological eradication rates are 56.65% - 80%, and the all-cause mortality rates are 11% - 41.6% (Dalfino et al., 2012; Durakovic et al., 2011; Grignolo et al., 2017; Li and Abad, 2020; Moni et al., 2020). Our results were also within the scope of previous studies (Dalfino et al., 2012; Durakovic et al., 2011; Grignolo et al., 2017; Li and Abad, 2020; Moni et al., 2020), and this wide range might reflect the different populations and clinical conditions studied. Furthermore, we have also compared the clinical effectiveness of CMS in the treatment of serious infections and sepsis in hematological diseases patients. A retrospective study showed that out of 28 hematological diseases patients with MDR Gram-negative bacteria infections, 86% of patients reported clinical cure, more than 80% patients achieved microbiological success and the 30-day mortality rate was 29%, yielding a slightly higher clinical response than in this study (Grignolo et al., 2017). These outcomes might be attributable to the fact that all the patients received therapy with high dose of CMS, while the patients in this study received CMS that did not exceed the loading dose. Importantly, the previous study included MDR-infected patients (Grignolo et al., 2017), whereas our study included sepsis patients, who typically have more severe disease and a poorer prognosis, which may have a significant impact on treatment response. Besides, the results of a matched pair analysis study showed that CMS was effective in treating sepsis caused by MDR-PA in patients with hematological diseases, with 76.9% of patients achieving clinical response and an all-cause mortality rate of 11% (Durakovic et al., 2011). A study conducted in our hospital on patients with infection during neutropenia in hematological diseases showed that the clinical effectiveness rate of ceftazidime-avibactam in the treatment of CRO infection was 65.8% (52/79), which is comparable to the clinical effectiveness rate of 62.96% (51/81) in this study. It is worth noting that all the patients in this study were sepsis patients, and the severity of infection was higher than that in previous study (Kong et al., 2022). Based on the above studies and our finding, CMS can be selected as an empirical treatment for hematological diseases patients diagnosed with sepsis.

Carbapenem resistant organisms pose a significant threat to the prognosis of patients with hematological diseases, significantly increasing their mortality rate. In this study, causative organisms were detected in 63 patients, more than 50% of whom were diagnosed with CRO infection. A synthesis of prior research indicated that CMS exhibited robust antibacterial activity against both MDR-Gram-negative bacteria and CRO infection, with bacteriological eradication rates ranging from 58.33% to 86.36% and 50% to 63.4%, respectively (Katip et al., 2023; Katip et al., 2021a; Tumbarello et al., 2013). In this study, 38 patients (60.23%) achieved bacteriological eradication, which strongly demonstrated the antibacterial activity of CMS. Furthermore, among the 33 patients who did not achieve bacteriological eradication or presumed not achieve bacteriological eradication, 24 had pulmonary infections. The combination of CMS intravenous with nebulized therapy has been demonstrated to enhance bacteriological eradication and clinical effectiveness in patients with pulmonary infections, further validation in large-scale studies is necessary to confirm these findings.

Available evidence suggests that high APACHE II score, high ΔSOFA score, past medical history, bloodstream infection, and septic shock are risk factors for clinical ineffective (Cheng et al., 2010; Li and Abad, 2020; Tumbarello et al., 2013). Consistently, our univariate and multivariate analysis indicated that high APACHE II score, the relapse or refractory of hematological diseases and septic shock was independently associated with a poor clinical effectiveness. Moreover, we found that clinical ineffective was significantly associated with higher Charlson comorbidity index and mechanical ventilation. These findings are not surprising, as evidence have confirmed that higher Charlson comorbidity index and invasive mechanical ventilation are associated with mortality in patients with hematologic malignancies admitted with serious infection (Awad et al., 2021; Zhen et al., 2023).

Several recent studies reported nephrotoxicity rates of 10.5% - 50%, these differences may be due to different study populations and dosages (Averbuch et al., 2013; Elefritz et al., 2017; Sadyrbaeva-Dolgova et al., 2022). It is worth noting that patients with hematological diseases are susceptible to renal injury due to various factors, including leukostasis, tumor lysis syndrome, nephrotoxic chemotherapy, and hypo perfusion due to sepsis. In the present study, the CMS demonstrated an acceptable safety profile despite the presence of the aforementioned risk factors, with a mere 18.52% of patients experiencing reversible renal insufficiency. The established RIFLE criteria were used to uniformly report possible colistin-related renal injuries. A retrospective study in hematology patients who received colistin-based therapy reported a severe (injury or failure) nephrotoxicity rate of 16%, requiring colistin discontinuation in 2 patients and colistin dose reduction in 1 patient (Grignolo et al., 2017). Similarly, the incidence of severe nephrotoxicity in this study was 12.34%. Only 1 patient met the “failure” criteria and 4 patients had an increase in Scr levels but did not reach the “failure” criteria discontinued using CMS. No patient required RRT treatment for more than 4 weeks due to renal insufficiency. Furthermore, the nephrotoxicity of CMS was usually dose-dependent and reversible, and the use of liposomes has been found to protect human embryonic kidney cells from concentration and time-dependent cytotoxicity (Mektrirat et al., 2023). Therefore, CMS is a relatively safe drug for patients with hematological diseases that developed serious infections. Previous studies have identified various predictors of CMS nephrotoxicity including older age, co-morbidities such as diabetes mellitus and systemic hypertension, and high dose of colistin (Elefritz et al., 2017; Katip et al., 2021b; Sadyrbaeva-Dolgova et al., 2022). However, univariate analysis in the present study failed to discern these associations, perhaps due to the different study population. Interestingly, we found that allo-HSCT have significant effects on renal insufficiency in hematological diseases patients, which may be related to receive chemotherapy (Nicolaysen, 2020). Furthermore, patients with AA and digestive tract infection also has a significant impact on renal insufficiency, and hematological diseases patients who have received allo-HSCT, have AA and digestive tract infections should be closely monitored. Additionally, 1 (1.23%) patient developed hepatic insufficiency, 1 (1.23%) developed cutaneous reaction, similar to another study (Grignolo et al., 2017), suggested that CMS therapy is safe for the management of sepsis in hematological diseases patients.

Our study has several limitations. Firstly, this single-center retrospective study relies on the record of events at the time of occurrence, which may lead to bias in the interpretation of clinical response and toxicity. Some patients were treated with CMS based on the prevalence of local drug-resistant bacteria and the patient’s current condition, which needs to be further validated by prospective studies. Secondly, our center is a specialist hematology hospital, and the characteristics of the patient population (such as high transplantation rate and high relapse rate) may affect the extrapolation of conclusions. Thirdly, due to the limited number of variables obtained from univariate analysis of renal insufficiency, further multivariate analysis was not conducted. Finally, a large multi-center prospective study was required to validate our results. Notwithstanding the aforementioned limitations, our study represents the inaugural investigation to ascertain the clinical outcomes and safety of CMS treatment for sepsis in Chinese patients with hematological diseases.

## 5 Conclusion

In summary, this study confirmed that CMS provided an effective treatment option for septic patients with hematologic diseases in China, particularly for high-risk populations such as post-transplant or neutropenia immunocompromised individuals in regions with high carbapenem resistance rates. CMS should be administered early during sepsis in cases with a high risk of CRO to control infections and improve patient outcomes. In addition, all patients in this study received intravenous CMS therapy. Future research should explore the potential synergistic effects of combining intravenous CMS with nebulized CMS for managing pulmonary infections.

## Data Availability

The original contributions presented in the study are included in the article/**Supplementary Material**. Further inquiries can be directed to the corresponding authors.
