# Supplementary material for: Colistimethate sodium is efficacious and safe for the management of sepsis in hematological diseases patients: a retrospective study in China
Source: Front Cell Infect Microbiol. 2025 Aug 13;15:1613414. doi: 10.3389/fcimb.2025.1613414 (PMC12380623; doi:10.3389/fcimb.2025.1613414)
Supplement: Supplementary file 1 [file Table1.docx]

**Supplementary Table 1** The differences between empirical and targeted therapy in terms of applicable conditions and basis for decision.

| **Type of therapy** | **Applicable conditions** | **Basis for decision** |
| --- | --- | --- |
| Empirical therapy | Patients who developed carbapenem-resistant organisms during the initial screening for drug-resistant bacteria, or who experienced infection breakthrough after broad-spectrum β-lactam/carbapenems treatment and progressed to sepsis | Local antimicrobial resistance data plus renal function |
| Targeted therapy | Confirmed in vitro susceptibility of the pathogen to colistimethate sodium | Microbiological evidence |
